# Effects of Dapagliflozin on Progression of CKD According to Different Rates of Pretrial eGFR Loss

**DOI:** 10.2215/CJN.0000000810

**Published:** 2025-09-09

**Authors:** Hiddo J.L. Heerspink, Glenn M. Chertow, Peter Rossing, Ricardo Correa-Rotter, C. David Sjöström, Robert D. Toto, David C. Wheeler, Niels Jongs

**Affiliations:** 1 Department of Clinical Pharmacy and Pharmacology, University Medical Center Groningen, University of Groningen, Groningen, The Netherlands; 2 Departments of Medicine, Epidemiology and Population Health, and Health Policy, Stanford University School of Medicine, Stanford, California; 3 Steno Diabetes Center Copenhagen, Herlev, Denmark; 4 Department of Clinical Medicine, University of Copenhagen, Copenhagen, Denmark; 5 Instituto Nacional de Ciencias Médicas y Nutrición Salvador Zubirán, Mexico-City, Mexico; 6 Late-stage Development, Cardiovascular, Renal, and Metabolism, BioPharmaceuticals R&D, AstraZeneca, Gothenburg, Sweden; 7 Department of Internal Medicine, UT Southwestern Medical Center, Dallas, Texas; 8 Department of Renal Medicine, University College London, London, United Kingdom

**Keywords:** epidemiology and outcomes, pharmacology, risk factors, SGLT2

## Abstract

**Key Points:**

- eGFR slope before a clinical trial may assist in selecting participants at risk of kidney outcomes in whom new therapies are needed.
- In a post hoc analysis of the Dapagliflozin and Prevention of Adverse Outcomes in Chronic Kidney Disease trial, we showed that the pretrial eGFR trajectory was associated with CKD progression.
- Patients who demonstrated rapid progression in advance of the trial experienced more pronounced benefits of dapagliflozin.

**Background:**

Identification of patients likely to experience substantial loss of eGFR is required to detect a kidney protective treatment effect in clinical trials; this is usually achieved by restricting inclusion of patients with elevated albuminuria. However, not all patients with elevated albuminuria show progressive eGFR loss. The eGFR slope before the trial may better predict whether patients experience loss in eGFR during the trial. We assessed the effect of dapagliflozin on eGFR slope according to patients' eGFR slope before enrollment (pretrial eGFR slope) in the Dapagliflozin and Prevention of Adverse Outcomes in Chronic Kidney Disease study.

**Methods:**

We recorded eGFR data for 2 or less to 2 years from electronic medical records for 4304 patients with CKD before enrollment in the Dapagliflozin and Prevention of Adverse Outcomes in Chronic Kidney Disease study. We used linear regression to estimate within-patient pre-enrollment eGFR trajectory. We evaluated the association of pre-enrollment eGFR trajectory with total and chronic eGFR slopes and a kidney composite end point. We also determined the degree to which pre-enrollment eGFR trajectory modified the effects of dapagliflozin.

**Results:**

Eight hundred and seventy (20% of the total cohort) patients with three or more historical eGFR measurements were evaluated (mean [SD] pre-enrollment eGFR slope: −6.1 [6.1] ml/min per 1.73 m^2^/year). The benefit of dapagliflozin in reducing total (*P* interaction 0.02) and chronic (*P* interaction 0.02) eGFR slopes was more pronounced in patients with steeper preinclusion eGFR trajectory (rapid progressors; eGFR slope <−5 ml/min per 1.73 m^2^/year), as was the benefit of dapagliflozin on the kidney composite end point (*P* interaction 0.02).

**Conclusions:**

Determination of pretrial eGFR trajectory may help to identify patients with CKD at higher and lower risks of progression and those more likely to benefit from targeted intervention.

**Clinical Trial Registry Name and Registration Number:**

NCT03036150

## Visual Abstract

**Figure:**
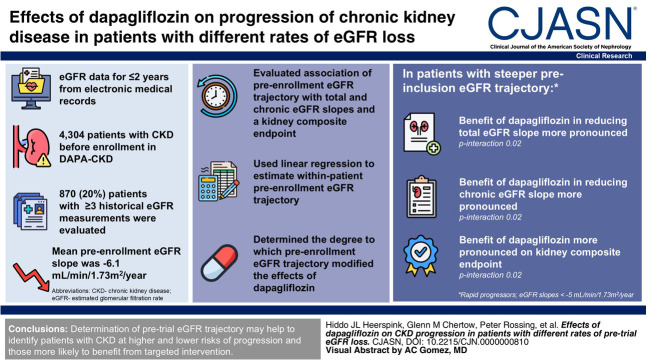

## Introduction

The Dapagliflozin and Prevention of Adverse Outcomes in Chronic Kidney Disease (DAPA-CKD) clinical trial showed that the sodium-glucose cotransporter 2 inhibitor (SGLT2i) dapagliflozin significantly reduced the risk of a primary composite kidney end point of kidney failure, sustained 50% decline in GFR and death due to kidney or cardiovascular causes.^1^ This end point is a composite of relative late events in the natural history of CKD. To obtain sufficient end points to achieve statistical power, the trial recruited patients likely to experience rapidly progressive disease by restricting the population to participants with increased albuminuria and a relatively low GFR. However, despite the selection of these patients, only approximately 10% of patients experienced a kidney event during the 2.4-year median follow-up time, an observation consistent with other kidney disease-focused clinical trials.^2–4^ Alternative strategies to enrich clinical trials for patients at risk for progressive kidney disease are worth considering, to increase trial efficiency or to expand the trial population to patients at earlier stages of kidney disease.

In most patients with CKD, kidney function deteriorates progressively over time. The rate of change in GFR before clinical trial enrollment (“pretrial GFR trajectory”) could be an alternative and intuitive approach to select patients for clinical trials. This would theoretically ensure enrollment of a population at high risk of CKD progression. However, this approach has not been tested in a phase 3 clinical trial setting.

A previous study compared the distribution of GFR trajectories in the dapagliflozin and placebo groups in the DAPA-CKD trial and suggested that the treatment effect of dapagliflozin in slowing GFR decline may be more pronounced in participants with a steep GFR decline in the first 2 weeks after randomization.^5^ To further explore this finding, the effect of dapagliflozin compared with placebo should ideally be determined in subgroups with varying GFR trajectories before enrollment.

The objectives of this *post hoc* analysis of the DAPA-CKD trial were therefore to assess the association of pretrial GFR trajectory with CKD progression. We subsequently determined whether the effects of dapagliflozin on CKD progression varied according to the pretrial eGFR trajectory.

## Methods

DAPA-CKD was a randomized, double-blind, placebo-controlled, multicenter clinical trial. The trial design, baseline characteristics, and primary results have been previously published.^1,6,7^ The DAPA-CKD trial was conducted at 386 sites in 21 countries from February 2017 to June 2020 and registered at ClinicalTrials.gov (identifier: NCT03036150). All participants provided written informed consent before any study-specific procedure commenced. The safety of participants in the trial was overseen by an independent Data Monitoring Committee. The trial was conducted according to the principles of the Declaration of Helsinki. Ethics committees at all participating centers approved the protocol.

### Participants

Eligible participants were aged 18 years or older with or without type 2 diabetes. They were required to have CKD with an eGFR between 25 and 75 ml/min per 1.73 m^2^ and urinary albumin-to-creatinine ratio (UACR) 200–5000 mg/g. Recruitment of patients with eGFR 60–75 ml/min per 1.73 m^2^ was limited to no more than 10% of trial participants. We required participants to be treated with a stable maximally tolerated dose of a renin-angiotensin-aldosterone system inhibitor (angiotensin-converting enzyme inhibitor or angiotensin receptor blocker) for ≥4 weeks, unless medically contraindicated. Key exclusion criteria included a documented diagnosis of type 1 diabetes, polycystic kidney disease, lupus nephritis, or antineutrophil cytoplasmic antibody-associated vasculitis. A complete list of inclusion and exclusion criteria and the trial protocol has been previously published.^7^

### Procedures

Participants were randomly assigned to dapagliflozin 10 mg daily or matching placebo, in accordance with the sequestered, fixed-randomization schedule, with the use of balanced blocks to ensure an approximate 1:1 ratio of the two regimens. Randomization was stratified by diabetes status and UACR (≤ or >1000 mg/g). We calculated eGFR using the CKD Epidemiology Collaboration equation and incorporated results from the equation as originally defined, including a term for self-reported race (Black versus non-Black race).^8^ Participants and all study personnel (except the Independent Data Monitoring Committee) were masked to treatment allocation. Investigators were requested to enter participant's serum creatinine values measured in local laboratories during 2 years before study enrollment in the electronic data capturing platform. We required that at least one measurement was collected between 4 and 8 months before study enrollment and another between 1 and 2 years. A maximum of four serum creatinine measurement per participant were recorded. After randomization, in-person study visits were performed at 2 weeks; at 2, 4, and 8 months; and at 4-month intervals thereafter. At each follow-up visit, study personnel recorded vital signs, obtained blood and urine samples, and recorded information on potential study end points, adverse events, concomitant therapies, and study drug adherence.

### End Points

The primary composite end point was time to ≥50% sustained decline in eGFR (confirmed by a second serum creatinine measurement after at least 28 days), onset of ESKD (defined as maintenance dialysis for at least 28 days, kidney transplantation, or eGFR <15 ml/min per 1.73 m^2^, confirmed by a second measurement after at least 28 days), or death from a kidney or cardiovascular cause. Secondary end points were time to a (*1*) kidney composite end point of ≥50% sustained decline in eGFR, ESKD, or death from kidney disease; (*2*) cardiovascular composite end point defined as hospitalization for heart failure or cardiovascular death; and (*3*) death from any cause. We also prespecified change in eGFR slope as exploratory efficacy end point. All events were adjudicated by a masked, independent Clinical Events Adjudication Committee; eGFR-based end points were not adjudicated.

### Statistical Analysis

To analyze the pretrial eGFR data, we selected participants whose pretrial eGFR trajectory was considered to be of sufficient quality. To this end, we fitted for each patient a within-participant linear regression model using the pretrial eGFR as the dependent variable and time as the independent variable. Pretrial eGFR trajectories from participants whose r-squared value was at least 0.5 and in whom the eGFR slope fell within the range of −25 to +5 ml/min per 1.73 m^2^/year were considered reliable and used for further analysis.

We summarized baseline characteristics categorized by pretrial eGFR trajectory using the following categories: rapid progression (pretrial eGFR trajectory <−5 ml/min per 1.73 m^2^ per year), modest progression (−5 to <−1 ml/min per 1.73 m^2^ per year), and nonprogressors (≥−1 ml/min per 1.73 m^2^ per year). Within these categories, we summarized numeric participant characteristics with an approximate symmetric distribution by their mean and SD. Characteristics with skewed distributions were reported by calculating their median (25th, 75th percentiles); discrete characteristics were reported as counts and proportions.

We analyzed the association between pretrial eGFR trajectory and on-treatment total and chronic eGFR slopes. The total slope was calculated from randomization to end-of-treatment and the chronic slope from the first on-treatment eGFR value until end of treatment, thus excluding the acute effect of dapagliflozin on eGFR. We fitted a two-slope mixed effects linear spline model, with a knot at 2 weeks and with correlated random intercepts and slopes for each participant over time, incorporating an unstructured covariance matrix. The mixed effects model included fixed effects for categories of pretrial eGFR trajectory, time, an interaction term for pretrial eGFR trajectory category by time, and baseline eGFR and the stratification factor (UACR ≤1000 mg/g or >1000 mg/g and type 2 diabetes status at baseline). We calculated the acute slope as the mean change in eGFR from baseline to week 2 and the chronic eGFR slope as the mean rate of change after week 2 until the last on-treatment visit. We did not impute missing follow-up eGFR values consistent with the main DAPA-CKD analyses.^1^ To assess whether the pretrial eGFR trajectory can be used to identify rapid progressors, we applied the model to the placebo group and determined the rate of eGFR decline in categories of pretrial eGFR trajectory as defined above, participants with baseline UACR ≤1000 mg/g or >1000 mg/g, and in subgroups based on the combination of UACR and pretrial eGFR trajectory. We also analyzed the association between pretrial eGFR trajectory as a continuous variable and eGFR during follow-up by using a restricted cubic spline on the independent variable to also allow for nonlinear associations.

We used Cox proportional hazards regression to estimate the hazard ratio (HR) and 95% confidence interval (CI) of dapagliflozin versus placebo on kidney and cardiovascular composite end points by pretrial eGFR trajectory categories. We adjusted models for baseline eGFR and stratified by UACR (UACR ≤1000 mg/g or >1000 mg/g) and type 2 diabetes status at baseline. We repeated this analysis adjusting for age, sex, baseline log-transformed UACR, cardiovascular disease history, and type 2 diabetes status. We also added the pretrial eGFR trajectory as a continuous variable to the Cox model and fitted the HR and 95% CI as a function of the pretrial eGFR trajectory. In this model, we calculated HRs relative to a preinclusion eGFR slope of 0 ml/min per 1.73 m^2^ per year using a restricted cubic spline with three knots at the 25th, 50th, and 75th percentiles of the pretrial eGFR slope. We used a Wald test to calculate a p-interaction value for interaction between preinclusion eGFR and treatment using the estimates from the model.

We conducted all statistical analyses using R version 4.4.1 (Vienna R statistics)

## Results

### Patient Disposition and Baseline Characteristics

A total of 870 patients (20% of all randomized participants) with at least three serum creatinine values before the start of the DAPA-CKD trial were analyzed. Of these patients, the mean number of pretrial serum creatinine measurements was 3.1 (SD 0.3; maximum 4), which were collected over a median duration of 1.04 years before inclusion (25th to 75th percentile 1.91 to 0.39).The mean pretrial eGFR trajectory was−6.1 ml/min per 1.73 m^2^/year, with a large between individual variability (2.5th to 97.5th percentile −19.5 to 4.0 ml/min per 1.73 m^2^/year).

Baseline characteristics of the 870 included participants were generally similar compared with the overall DAPA-CKD cohort (Supplemental Table 1). Participants with a pretrial eGFR trajectory <−5 ml/min per 1.73 m^2^/year (rapid progressors) had a lower hemoglobin, higher UACR, and were more likely to have cardiovascular disease compared with those with an annual eGFR slope between−1 and−5 ml/min per 1.73 m^2^ (moderate progressors) or ≥−1 ml/min per 1.73 m^2^ (nonprogressors; Table 1).

**Table 1:** Baseline characteristics

Characteristics	Pretrial eGFR Slope (ml/min/1.73 m^2^ per yr)			
	<−5 (*n*=487)	≥−5 to <−1 (*n*=230)	≥−1 (*n*=153)	Total (*N*=870)
Pretrial eGFR slope, mean (SD)	−10.3 (4.5)	−3.1 (1.1)	2.4 (1.5)	−6.1 (6.1)
Dapagliflozin assignment, *n* (%)	239 (49.1)	116 (50.4)	57 (37.3)	412 (47.4)
Age, yr, mean (SD)	62 (12)	62 (12)	62 (12)	62 (12)
Sex, female, *n* (%)	135 (28)	63 (27)	45 (29)	243 (28)
**Race, *n* (%)** ^a^				
Asian	216 (44)	99 (43)	53 (35)	368 (42)
Black or African American	26 (5)	7 (3)	5 (3)	38 (4)
Others	18 (4)	8 (4)	6 (4)	32 (4)
White	227 (47)	116 (50)	89 (58)	432 (50)
Weight, kg, mean (SD)^b^	82.4 (21.2)	82.4 (20.1)	83.6 (21.2)	82.6 (20.9)
BMI, kg/m^2^, mean (SD)^c^	29.5 (6.5)	29.4 (5.9)	29.9 (6.4)	29.5 (6.3)
Current smoker, *n* (%)	77 (16)	29 (13)	19 (12)	125 (14)
Systolic BP, mm Hg, mean (SD)	137.1 (18.2)	136.0 (17.3)	136.8 (18.0)	136.8 (17.9)
Diastolic BP, mm Hg, mean (SD)	76.2 (11.1)	77.9 (10.7)	76.6 (10.6)	76.7 (10.9)
HbA1c, (%), mean (SD)^d^	6.9 (1.5)	6.6 (1.4)	6.8 (1.4)	6.8 (1.5)
eGFR, ml/min per 1.73 m^2^, mean (SD)	39.8 (11.1)	37.6 (10.3)	42.0 (11.7)	39.6 (11.1)
**eGFR stages, ml/min per 1.73 m** ^**2**^ **, *n* (%)**				
≥60	25 (5)	10 (4)	14 (9)	49 (6)
45–<60	111 (23)	44 (19)	47 (31)	202 (23)
30–<45	255 (52)	116 (50)	70 (46)	441 (51)
<30	96 (20)	60 (26)	22 (14)	178 (21)
Hemoglobin, g/L, mean (SD)^e^	125.5 (16.5)	131.4 (17.9)	130.6 (18.1)	128.0 (17.3)
Potassium, mmol/L, mean (SD)^f^	4.7 (0.5)	4.7 (0.6)	4.6 (0.5)	4.7 (0.5)
UACR, mg/g, median (25th, 75th percentile)	1161.5 (548.0, 2416.5)	904.2 (484.2, 1810.9)	965.0 (519.5, 1528.5)	1023.2 (519.8, 1982.8)
UACR >1000, *n* (%)	272 (56)	102 (44)	70 (46)	444 (51)
Type 2 diabetes, *n* (%)	329 (68)	135 (59)	97 (63)	561 (65)
Cardiovascular disease, *n* (%)	181 (37)	83 (36)	51 (33)	315 (36)
Heart failure, *n* (%)	44 (9)	23 (10)	13 (9)	80 (9)
**Previous medication, *n* (%)**				
ACEi, *n* (%)	135 (28)	68 (30)	50 (33)	253 (29)
ARB, *n* (%)	339 (70)	161 (70)	101 (66)	601 (69)
Diuretics, *n* (%)	228 (47)	99 (43)	78 (51)	405 (47)
Statins, *n* (%)	327 (67)	172 (75)	116 (76)	615 (71)
Antithrombotic agents, *n* (%)	244 (50)	112 (49)	71 (46)	427 (49)

ACEi, angiotensin-converting enzyme inhibitor; ARB, angiotensin-receptor blocker; IQR, interquartile range; UACR, urinary albumin-to-creatinine ratio.

a Race was self-reported.

b <−5, *n*=486; total, *n*=869.

c <−5, *n*=486; ≥−5 to <−1, *n*=229; total, *n*=868.

d ≥−5 to <−1, *n*=229; ≥−1, *n*=152; total, *n*=868.

e <−5, *n*=486; ≥−1, *n*=152; total, *n*=868.

f <−5, *n*=485; total, *n*=868.

### Rates of eGFR Decline during Follow-Up According to Pretrial eGFR Trajectory and UACR

**Figure 1:**
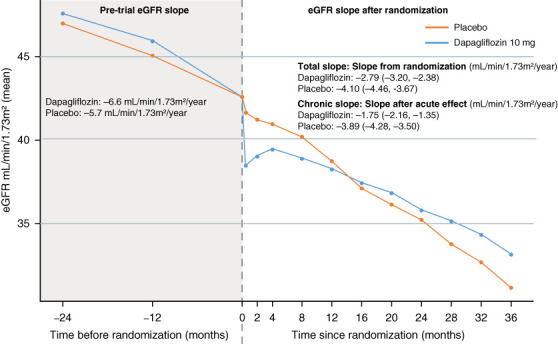
**Rate of eGFR trajectories in the placebo and dapagliflozin treatment groups before entry into the DAPA-CKD trial (gray shaded area) and after randomization into the DAPA-CKD trial (white area).** Values presented as mean (95% CI). CI, confidence interval; DAPA-CKD, Dapagliflozin and Prevention of Adverse Outcomes in Chronic Kidney Disease.

**Figure 2:**
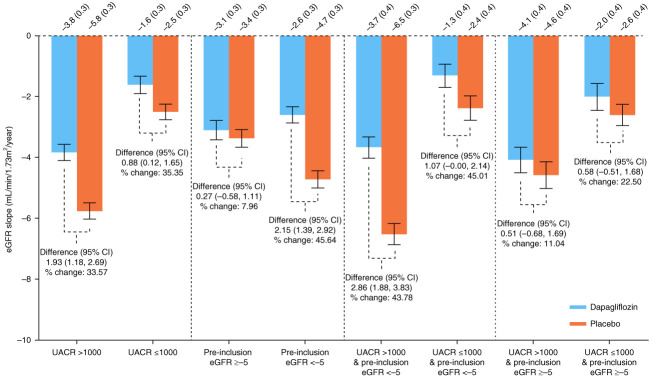
**Total eGFR slope in the placebo (orange) and dapagliflozin groups (blue) in subgroups defined by the pretrial eGFR slope and baseline albuminuria.** Data represented as mean (SD) unless otherwise indicated. UACR, urinary albumin-to-creatinine ratio.

After enrollment into the trial, the annual mean eGFR slope in the placebo group was−4.1 (95% CI, −4.5 to −3.7) ml/min per 1.73 m^2^ (Figure 1). When participants assigned to the placebo group were stratified by the pretrial eGFR decline, the mean eGFR slope in participants with a pretrial eGFR trajectory ≤−5 and >−5 ml/min per 1.73 m^2^/year was −4.7 (SEM 0.3) and −3.4 (SEM 0.3) ml/min per 1.73 m^2^/year, respectively. When placebo-treated participants were stratified based on baseline UACR ≤1000 and >1000 mg/g, the mean eGFR slope during follow-up was −2.5 (SEM 0.3) and−5.8 (SEM 0.3) ml/min per 1.73 m^2^ per year, respectively. When stratifying on both the pretrial eGFR trajectory and baseline UACR, those with a pretrial eGFR trajectory ≤−5 ml/min per 1.73 m^2^ per year and UACR >1000 mg/g had the steepest eGFR slope (Figure 2). In a multivariable model, baseline eGFR and UACR as well as pretrial eGFR trajectory were independently associated with eGFR slope during the trial (Table 2 and Supplemental Table 2).

**Table 2:** Baseline characteristics associated with total eGFR slope

Characteristics	Univariable		Multivariable	
	Coefficient (95% CI)	*P* Value	Coefficient	*P* Value
Pretrial eGFR slope, per ml/min per 1.73 m^2^ per yr	0.38 (0.31 to 0.45)	<0.001	0.12 (0.06 to 0.17)	<0.001
Age, per yr	0.02 (-0.00 to 0.03)	0.11	0.04 (0.01 to 0.07)	0.01
Sex (female)	−0.21 (−0.67 to 0.26)	0.39	−0.73 (−1.51 to 0.06)	0.07
Systolic BP, per mm Hg	−0.04 (−0.06 to −0.03)	<0.001	−0.00 (−0.02 to 0.02)	0.91
eGFR, per ml/min per 1.73 m^2^	−0.04 (−0.06 to −0.03)	<0.001	−0.07 (−0.10 to −0.03)	<0.001
UACR, per log mg/g	−2.49 (−2.73 to −2.25)	<0.001	−2.01 (−2.46 to −1.57)	<0.001

Multivariate linear mixed effects model is adjusted for the baseline covariates. There were no missing values in the baseline characteristics.

UACR, urinary albumin-to-creatinine ratio.

### Effect of Dapagliflozin on Rates of eGFR Decline and Clinical End Points According to Pretrial eGFR Trajectory

**Figure 3:**
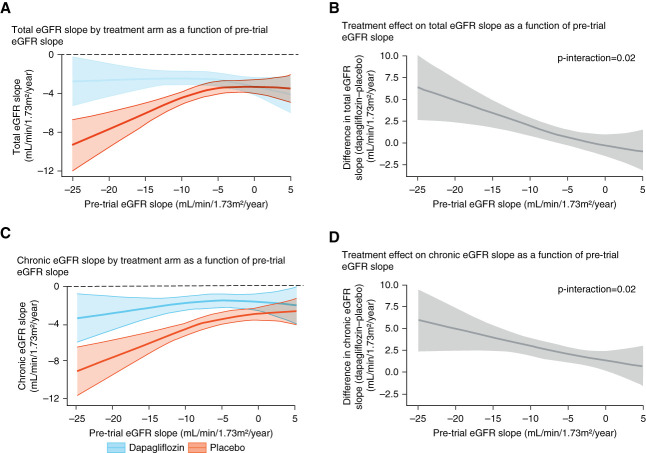
**Impact and treatment effect on eGFR slope as a function of continuous pre-trial eGFR slope.** (A) Total eGFR slope as a function of the pre-trial eGFR slope. (B) Treatment effect on the total eGFR slope as a function of the pre-trial eGFR slope. (C) Chronic eGFR slope as a function of the pre-trial eGFR slope. (D) Treatment effect on the chronic eGFR slope as a function of the pre-trial eGFR slope.

The total eGFR slope during double blind treatment in the DAPA-CKD trial was −2.8 (SEM 0.2) ml/min per 1.73 m^2^/year in the dapagliflozin group and−4.1 (SEM 0.2) ml/min per 1.73 m^2^/year in the placebo group, corresponding to a between-group difference of 1.3 ml/min per 1.73 m^2^/year (95% CI, 0.7 to 1.9; Figure 2). When the eGFR slope was calculated from the first on-treatment visit, thus excluding the acute effect of dapagliflozin on eGFR, the between group difference in the chronic eGFR slope was 2.1 (95% CI, 1.6 to 2.7) ml/min per 1.73 m^2^/year. The benefit of dapagliflozin in reducing the total (*P* interaction 0.02) and chronic (*P* interaction 0.02) eGFR slopes was more pronounced in patients with a steeper pretrial eGFR trajectory (*i.e*., in rapid progressors; Figure 3). Among participants with a pretrial eGFR slope <−5 ml/min per 1.73 m^2^ per year, the mean rates of eGFR decline from randomization in the dapagliflozin and placebo groups were 2.6 (SEM 0.3) ml/min per 1.73 m^2^/year and 4.7 (SEM 0.3) ml/min per 1.73 m^2^/year, respectively, corresponding to a between group difference of 2.2 ml/min per 1.73 m^2^/year (95% CI, 1.4 to 2.9). By contrast, in participants with a pretrial eGFR trajectory >−5 ml/min per 1.73 m^2^/year, rates of eGFR decline during the trial in the dapagliflozin and placebo groups were−3.1 (SEM 0.3) and−3.4 (SEM 0.3) ml/min per 1.73 m^2^/year, respectively, corresponding to a between group difference of 0.3 (95% CI, −0.6 to 1.1) ml/min per 1.73 m^2^/year (Figure 1). Similar results were obtained when these analyses were adjusted for baseline UACR (Supplemental Figure 1).

**Figure 4:**
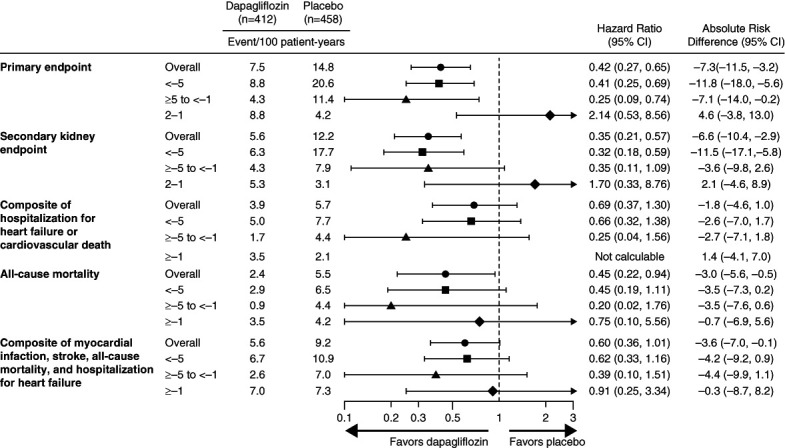
Relative and absolute risk changes of dapagliflozin versus placebo on kidney, cardiovascular, and mortality outcomes in participant subgroups defined by pretrial eGFR slope categories.

During follow-up, 99, 42, and 35 kidney composite end points, cardiovascular composite end points, and deaths from any cause were recorded. Rates of these clinical end points were markedly higher among rapid progressors compared with moderate or slow/nonprogressors (Figure 4). Dapagliflozin compared with placebo reduced the risks of the kidney composite end point among rapid progressors (HR, 0.41 [95% CI, 0.25 to 0.69]) and moderate progressors (HR, 0.25 [95% CI, 0.09 to 0.74]) but not among slow or nonprogressors (HR 2.14 [95% CI, 0.53 to 8.56]); *P* interaction 0.02; Figure 4). Overall, HRs for the effects of dapagliflozin compared with placebo on the risks of the cardiovascular composite end point and death from any cause outcomes were 0.69 (95% CI, 0.37 to 1.30) and 0.45 (95% CI, 0.22 to 0.94) with no evidence of effect modification by the pretrial eGFR trajectory (*P* interaction 0.46 and 0.52, respectively).

## Discussion

In this *post hoc* analysis of 870 DAPA-CKD participants with three or more determinants of eGFR within 2 years of trial enrollment, there was wide variation in rates of eGFR decline. Overall, more than half of patients showed a pretrial eGFR trajectory of 5 or higher ml/min per 1.73 m^2^/year, whereas the eGFR slope was more than −1 ml/min per 1.73 m^2^ in fewer than one in five participants. Pretrial eGFR trajectory was associated with on-trial eGFR total and chronic slopes and the kidney composite end point. Moreover, patients who demonstrated rapid progression in advance of the trial experienced more pronounced benefits of dapagliflozin suggesting that the pretrial eGFR trajectory may aid in identifying participants more likely to experience kidney protection with dapagliflozin.

Although the UACR is a well-established risk marker to enrich a clinical trial for participants at high risk of CKD progression, there remains a fraction of patients with moderate or severely increased albuminuria in whom eGFR remains stable during the relatively short duration of a clinical trial; conversely, there are patients with more modest levels of albuminuria who exhibit rapid progression. This highlights the need to identify alternative strategies to identify participants at high risk of kidney failure. In the DAPA-CKD trial, the eGFR decline during the trial was more pronounced in fast progressors and even more so in fast progressors with UACR >1000 mg/g. These data suggest a role for the pretrial eGFR trajectory as a risk enrichment criterion in future clinical trials.

Patients with a rapid loss of kidney function are at highest need for treatment intensification. The benefits of dapagliflozin in reducing the eGFR slope were most pronounced in participants with the steepest pretrial eGFR trajectory, those with a pretrial eGFR slope <−5 ml/min per 1.73 m^2^ per year and UACR of at least 1000 mg/g, suggesting that both the pretrial eGFR slope and baseline albuminuria confer complimentary information to identify patients in whom dapagliflozin may exert a pronounced effect in attenuating eGFR decline. These data support previous conclusions that SGLT2is exert more potent relative treatment effects in rapid progressors rather than in moderate or slower progressors.^5^

In addition to reducing the risk of CKD progression, dapagliflozin also reduced the risk of hospitalization due to heart failure and cardiovascular death as well as all-cause mortality. These benefits of dapagliflozin on heart failure and mortality were consistent regardless of the pretrial eGFR slope. In other words, while the effect of dapagliflozin on attenuating eGFR decline was more pronounced in fast progressors, those who progress more slowly also derive cardiovascular and mortality benefits. The relatively short 2.4-year median follow-up of the DAPA-CKD trial precludes assessment of kidney benefits over longer time horizons. Patients with slower rates of progression might derive kidney benefits over an extended period as recently demonstrated in the long-term follow-up observational period of the The Study of Heart and Kidney Protection with Empagliflozin trial.

Other prospective clinical trials have used the pretrial eGFR trajectory to enrich the population for participants at risk of kidney disease progression. In the Preventing Early Renal Loss in Diabetes trial, participants with type 1 diabetes and CKD could be enrolled based on either elevated albuminuria or a history of a rapid eGFR decline.^9^ The trial recruited 394 participants based on the albuminuria criterion and 124 participants based on the eGFR decline criterion. Comparison of the selection criteria to assess which parameter most effectively identified fast progressors showed that participants with elevated albuminuria experienced a faster rate of eGFR loss compared with participants enrolled based on the pretrial eGFR trajectory. This led the authors to conclude that the pretrial eGFR trajectory is a less powerful selection tool than albuminuria to identify patients at high risk of rapid kidney disease progression.^9^ The Effect of CANagliflozin in type 2 diabetic PatIents with micrOalbuminurIa in JapaNEse population (CANPIONE) trial, an open-label trial of the SGLT2i canagliflozin, enrolled patients with type 2 diabetes and microalbuminuria and included a preintervention period of 24 weeks during which eGFR was measured in a central laboratory, supplemented by eGFR data collected from electronic medical records up to 3 years before study enrollment.^10^ The results demonstrated that 52 weeks of treatment with canagliflozin reduced the rates of eGFR decline relative to the pretrial eGFR decline. This benefit of canagliflozin was detected in only 96 trial participants at early CKD stages who generally showed a modest decline in eGFR. Importantly, similarly to this study, a *post hoc* analysis suggested that the benefit of canagliflozin may be more pronounced in the subgroup with the steepest decline in pretrial eGFR. These data suggest that the use of a pretrial eGFR slope may be an efficient approach to detect treatment effects in participants with type 2 diabetes and early stages of CKD.

Important questions remain regarding the optimal operationalization of the pretrial eGFR trajectory in future trials. Aspects regarding the timing and frequency of eGFR measurements and the duration of the pretrial period remain unclear. The Effect of CANagliflozin in type 2 diabetic PatIents with micrOalbuminurIa in JapaNEse population trial showed that the between individual variability in the pretrial eGFR trajectory collected during a 6-month long pretrial “run-in” period with monthly eGFR measurements under standardized condition is suboptimal compared with using eGFR data from electronic medical records collected over 3 years before trial enrollment. However, whether more frequent serum creatinine measurements during a pretrial run-in period improves the precision of the pretrial eGFR trajectory—perhaps facilitated by the use of innovative technologies that enable participants to collect blood and measure eGFR remotely—is unclear.^10^ Additional studies are therefore needed. These studies should also assess the role of intercurrent events, such as AKI episodes and initiation of interventions with acute effect on eGFR, on the precision of the pretrial eGFR trajectory.

These data have implications for the design of future trials in CKD. Establishing the pretrial eGFR trajectory may aid in selection of patients with the steepest eGFR decline during the trial and highest risk of CKD progression. This would increase statistical power and decrease sample size requirements, assuming a consistent treatment effect, but decreases generalizability as it excludes newly diagnosed participants and/or patients where historical data are unavailable. These data also confirm previous studies, suggesting that the clinical benefit of SGLT2is on CKD progression depend, at least partly, on the underlying rate of eGFR decline with a more pronounced effect among fast progressors.^5^ Whether this is a specific feature of SGLT2is or applies to other kidney protective interventions requires further study.

This study has several limitations. First, the DAPA-CKD trial enrolled participants with UACR above 200 mg/g. Whether the pretrial eGFR trajectory would be more or less informative among patients with lower levels of albuminuria remains to be determined. We recorded a maximum of four eGFR datapoints during 2 years before randomization, despite the fact that some participants had more eGFR measurements present in their electronic medical records. Had we included more than four eGFR datapoints, we could have enhanced the precision of the pretrial eGFR trajectory. This may have affected the precision of the pretrial eGFR slope. In addition, despite this being one of the largest studies on this topic to date, we were only able to record the pretrial eGFR slope in a random selection of 870 participants potentially decreasing the precision of our pretrial eGFR slope effect estimates.

In conclusion, among patients with CKD with and without diabetes and albuminuria, the pretrial eGFR trajectory was independently associated with CKD progression, whether considering total eGFR slope, chronic eGFR slope, or the kidney composite end point; patients with a steeper eGFR decline in the 2 years preceding the trial experienced more pronounced benefits of dapagliflozin. Incorporating historical eGFR trajectory into inclusion criteria could refine and enhance future trials aiming to preserve kidney function and reduce the incidence and severity of associated complications.

## Supplementary Material

## Data Availability

Original data generated for the study will be made available upon reasonable request to the corresponding author. Data Type: Clinical Trial Data; Research Protocols. Reason for Restricted Access: Data underlying the findings described in this manuscript may be obtained in accordance with AstraZeneca's data sharing policy described at: https://astrazenecagrouptrials.pharmacm.com/ST/Submission/Disclosure Disclosure Data for studies directly listed on Vivli can be requested through Vivli at www.vivli.org. Data for studies not listed on Vivli could be requested through Vivli at https://vivli.org/members/enquiries-about-studies-not-listed-on-the-vivli-platform/. AstraZeneca Vivli member page is also available outlining further details: https://vivli.org/ourmember/astrazeneca.

## Declarative Statements

This study includes clinical experimentation and received Institutional Review Board or Ethics Committee approval. All patients provided written informed consent. This study includes clinical experimentation and complies with the Declaration of Helsinki.

## Supplemental Material

Supplemental Table 1. Baseline characteristics stratified by treatment allocation and pretrial eGFR trajectory.

Supplemental Table 2. On-treatment total eGFR slope trajectories by pretrial enrollment eGFR slope trajectories.

Supplemental Figure 1. Between-group differences in on-trial total eGFR slope as a function of the pretrial eGFR slope.
