# Supplementary material for: Effects of Dapagliflozin on Progression of CKD According to Different Rates of Pretrial eGFR Loss
Source: Clin J Am Soc Nephrol. 2025 Sep 9;20(11):1527–35. doi: 10.2215/CJN.0000000810 (PMC12614848; doi:10.2215/CJN.0000000810)
Supplement: Supplementary file 1 [file cjasn-20-1527-s001.pdf]

ASN American Society of Nephrology | JASN Journal of the American Society of Nephrology | KIDNEY360 Accessing Our World From Every Angle | CJASN Clinical Journal of the American Society of Nephrology

ASN Journal Disclosure Form

As per ASN journal policy, I have disclosed any financial relationships or commitments I have held in the past 36 months as included below. I have listed my Current Employer below to indicate there is a relationship requiring disclosure. If no relationship exists, my Current Employer is not listed.

G. Chertow reports the following:
Employer: Stanford University School of Medicine; Consultancy: Akebia, Alebund, Ardelyx, AstraZeneca, CalciMedica, Miromatrix, Panoramic, Sanifit, Toku, Unicycive, Vertex; Ownership Interest: Ardelyx, CloudCath, Durect, Eliaz Therapeutics, Outset, Renibus, Unicycive; Research Funding: NIDDK, NIAID, CSL Behring; Advisory or Leadership Role: Board of Directors, Satellite Healthcare, Co-Editor, Brenner & Rector's The Kidney (Elsevier); and Other Interests or Relationships: DSMB service: NIDDK, George Institute, Aethlon, Bayer, Mineralys, ReCor.

I understand that the information above will be published within the journal article, if accepted, and that failure to comply and/or to accurately and completely report the potential financial conflicts of interest could lead to the following: 1) Prior to publication, article rejection, or 2) Post-publication, sanctions ranging from, but not limited to, issuing a correction, reporting the inaccurate information to the authors' institution, banning authors from submitting work to ASN journals for varying lengths of time, and/or retraction of the published work.

Name: Glenn M. Chertow
Manuscript ID: CJASN-2025-000773R2
Manuscript Title: Effects of dapagliflozin on CKD progression in patients with different rates of eGFR loss
Date of Completion: August 26, 2025
Disclosure Updated Date: January 22, 2025

ASN Journal Disclosure Form

As per ASN journal policy, I have disclosed any financial relationships or commitments I have held in the past 36 months as included below. I have listed my Current Employer below to indicate there is a relationship requiring disclosure. If no relationship exists, my Current Employer is not listed.

R. Correa-Rotter reports the following:
Employer: Instituto Nacional de Ciencias Médicas y Nutrición Salvador Zubiran, MEXICO and; Universidad Nacional Autónoma de México, MEXICO; Consultancy: Astra Zeneca, GSK, Boheringer Ingelheim, Bayer, Chinook, Novonordisk; Research Funding: Astra Zeneca, Novonordisk, Roche, Chinook, GSK; Honoraria: Amgen, Astra Zeneca, Boehringer Ingelheim, Sanofi, Bayer, Amgen; Advisory or Leadership Role: Membership Steering Committee of DAPA CKD, Astra Zeneca.; National Leader ASCEND study, GSK; National Leader FLOW study, Novonordisk, membership steering Committee FINE-REAL, Bayer.. Editorial Board Nefrologia Latinoamericana, Revista de Investigación Clinica, American Journal of Kidney Diseases. y Associate Editor: Blood Purification Associate Editor. Member of the Steering Committee of the World Kidney Day organization.; Speakers Bureau: Amgen, Astra Zeneca, Boehringer Ingelheim, Abbvie, Sanofi, Bayer, Novonordisk; and Other Interests or Relationships: Member of ASN, of International Society of Nephrology; Member of National Kidney Foundation; Member Mexican Institute for Research in Nephrology; Member Latin American Society of Nephrology and Hypertension; Member EDTA/ERA.

I understand that the information above will be published within the journal article, if accepted, and that failure to comply and/or to accurately and completely report the potential financial conflicts of interest could lead to the following: 1) Prior to publication, article rejection, or 2) Post-publication, sanctions ranging from, but not limited to, issuing a correction, reporting the inaccurate information to the authors' institution, banning authors from submitting work to ASN journals for varying lengths of time, and/or retraction of the published work.

Name: Ricardo Correa-Rotter
Manuscript ID: CJASN-2025-000773R2
Manuscript Title: Effects of dapagliflozin on CKD progression in patients with different rates of eGFR loss.
Date of Completion: July 29, 2025
Disclosure Updated Date: November 9, 2024

ASN Journal Disclosure Form

As per ASN journal policy, I have disclosed any financial relationships or commitments I have held in the past 36 months as included below. I have listed my Current Employer below to indicate there is a relationship requiring disclosure. If no relationship exists, my Current Employer is not listed.

H. Heerspink reports the following:
Employer: University Medical Center Groningen; Consultancy: Ongoing consultancy agreements with AstraZeneca, Alexion, Bayer, Boehringer Ingelheim, Biocity Therapeutic, Dimerix, Eli-Lilly, Gilead, Janssen, Novartis, NovoNordisk, Roche, Travere Therapeutics; Research Funding: AstraZeneca, Bayer, Boehringer Ingelheim, NovoNordisk and Janssen research support (grant funding directed to employer); Honoraria: Lecture fees from AstraZeneca and NovoNordisk; and Speakers Bureau: AstraZeneca, Bayer, Novo Nordisk.

I understand that the information above will be published within the journal article, if accepted, and that failure to comply and/or to accurately and completely report the potential financial conflicts of interest could lead to the following: 1) Prior to publication, article rejection, or 2) Post-publication, sanctions ranging from, but not limited to, issuing a correction, reporting the inaccurate information to the authors' institution, banning authors from submitting work to ASN journals for varying lengths of time, and/or retraction of the published work.

Name: Hiddo Jan L. Heerspink
Manuscript ID: CJASN-2025-000773R1
Manuscript Title: Effects of dapagliflozin on CKD progression in patients with different rates of eGFR loss
Date of Completion: June 22, 2025
Disclosure Updated Date: June 7, 2025

ASN American Society of Nephrology | JASN Journal of the American Society of Nephrology | KIDNEY360 Accessing Our World From Every Angle | CJASN Clinical Journal of the American Society of Nephrology

ASN Journal Disclosure Form

As per ASN journal policy, I have disclosed any financial relationships or commitments I have held in the past 36 months as included below. I have listed my Current Employer below to indicate there is a relationship requiring disclosure. If no relationship exists, my Current Employer is not listed.

N. Jongs reports the following:
Employer: UMCG; and Speakers Bureau: AstraZeneca.

I understand that the information above will be published within the journal article, if accepted, and that failure to comply and/or to accurately and completely report the potential financial conflicts of interest could lead to the following: 1) Prior to publication, article rejection, or 2) Post-publication, sanctions ranging from, but not limited to, issuing a correction, reporting the inaccurate information to the authors' institution, banning authors from submitting work to ASN journals for varying lengths of time, and/or retraction of the published work.

Name: Niels Jongs
Manuscript ID: CJASN-2025-000773R1
Manuscript Title: Effects of dapagliflozin on CKD progression in patients with different rates of eGFR loss
Date of Completion: June 23, 2025
Disclosure Updated Date: May 22, 2025

ASN
American Society of Nephrology
JASN
Journal of the American Society of Nephrology
KIDNEY360
Accessing Our World From Every Angle
CJASN
Clinical Journal of the American Society of Nephrology

ASN Journal Disclosure Form

As per ASN journal policy, I have disclosed any financial relationships or commitments I have held in the past 36 months as included below. I have listed my Current Employer below to indicate there is a relationship requiring disclosure. If no relationship exists, my Current Employer is not listed.

P. Rossing reports the following:
Employer: Steno Diabetes Center Copenhagen; Research Funding: Novo Nordisk , AstraZeneca, Bayer, Lexicon Pharma; Honoraria: Boehringer Ingelheim, AstraZeneca, Abbott, Novo Nordisk, all honoraria to institution; and Advisory or Leadership Role: Astra Zeneca Bayer , Novo Nordisk, Gilead all honoraria to institution.

I understand that the information above will be published within the journal article, if accepted, and that failure to comply and/or to accurately and completely report the potential financial conflicts of interest could lead to the following: 1) Prior to publication, article rejection, or 2) Post-publication, sanctions ranging from, but not limited to, issuing a correction, reporting the inaccurate information to the authors' institution, banning authors from submitting work to ASN journals for varying lengths of time, and/or retraction of the published work.

Name: Peter Rossing
Manuscript ID: CJASN-2025-000773R1
Manuscript Title: Effects of dapagliflozin on CKD progression in patients with different rates of eGFR loss,"
Date of Completion: July 4, 2025
Disclosure Updated Date: March 19, 2025

ASN American Society of Nephrology
JASN Journal of the American Society of Nephrology
KIDNEY360 Accessing Our World From Every Angle
CJASN Clinical Journal of the American Society of Nephrology

ASN Journal Disclosure Form

As per ASN journal policy, I have disclosed any financial relationships or commitments I have held in the past 36 months as included below. I have listed my Current Employer below to indicate there is a relationship requiring disclosure. If no relationship exists, my Current Employer is not listed.

D. Sjostrom reports the following:
Employer: AstraZeneca LP; and Ownership Interest: AstraZeneca LP.

I understand that the information above will be published within the journal article, if accepted, and that failure to comply and/or to accurately and completely report the potential financial conflicts of interest could lead to the following: 1) Prior to publication, article rejection, or 2) Post-publication, sanctions ranging from, but not limited to, issuing a correction, reporting the inaccurate information to the authors' institution, banning authors from submitting work to ASN journals for varying lengths of time, and/or retraction of the published work.

Name: David Sjostrom
Manuscript ID: CJASN-2025-000773R1
Manuscript Title: Effects of dapagliflozin on CKD progression in patients with different rates of eGFR loss
Date of Completion: July 1, 2025
Disclosure Updated Date: June 8, 2025

ASN
American Society of Nephrology
JASN
Journal of the American Society of Nephrology
KIDNEY360
Accessing Our World From Every Angle
CJASN
Clinical Journal of the American Society of Nephrology

ASN Journal Disclosure Form

As per ASN journal policy, I have disclosed any financial relationships or commitments I have held in the past 36 months as included below. I have listed my Current Employer below to indicate there is a relationship requiring disclosure. If no relationship exists, my Current Employer is not listed.

R. Toto reports the following:
Employer: University of Texas Southwestern Medical Center; Consultancy: Astra-Zeneca, Boehringer-Ingelheim, Alnylm Pharma, EpoK Pharma; Research Funding: NIH; Vertex pharma; Honoraria: Astra-Zeneca, Alnylam Pharma, Novo Nordisk; and Advisory or Leadership Role: Astra-Zeneca, Boehringer-Ingelheim,Novo Nordisk, EpoK.

I understand that the information above will be published within the journal article, if accepted, and that failure to comply and/or to accurately and completely report the potential financial conflicts of interest could lead to the following: 1) Prior to publication, article rejection, or 2) Post-publication, sanctions ranging from, but not limited to, issuing a correction, reporting the inaccurate information to the authors' institution, banning authors from submitting work to ASN journals for varying lengths of time, and/or retraction of the published work.

Name: Robert D. Toto
Manuscript ID: CJASN-2025-000773R2
Manuscript Title: Effects of dapagliflozin on CKD progression in patients with different rates of eGFR loss
Date of Completion: July 30, 2025
Disclosure Updated Date: July 30, 2025

ASN
American Society of Nephrology
JASN
Journal of the American Society of Nephrology
KIDNEY360
Accessing Our World From Every Angle
CJASN
Clinical Journal of the American Society of Nephrology

ASN Journal Disclosure Form

As per ASN journal policy, I have disclosed any financial relationships or commitments I have held in the past 36 months as included below. I have listed my Current Employer below to indicate there is a relationship requiring disclosure. If no relationship exists, my Current Employer is not listed.

D. Wheeler reports the following:
Employer: University College London; Consultancy: Fees from: Astellas, AstraZeneca, Bayer, Boehringer Ingelheim, Dimerix, Eledon, Galderma, George Clinical, Gilead, GlaxoSmithKline, Janssen, Merck Sharp and Dohme, Mineralys, Pathalys, Pfizer, ProKidney, Sana, Silence, Synthekine, Tricida, Vifor, Vertex, Zydus for Talks, Advisory Boards, Trial Committees and Consultancy; Research Funding: National Institute for Health Research; Kidney Research UK; Honoraria: Astellas, AstraZeneca, Bayer, Boehringer Ingelheim, Eledon, Galderma, George Clinical, GlaxoSmithKline, Merck Sharp and Dohme, Mineralys, Pathalys, ProKidney, Sana, Silence, Synthekine, Vifor, Vertex,; Advisory or Leadership Role: National Institute of Health Research Clinical Lead for Renal Disorders.; International society of Nephrology; American Journal of Kidney Disease; and Speakers Bureau: AstraZeneca, Astellas.

I understand that the information above will be published within the journal article, if accepted, and that failure to comply and/or to accurately and completely report the potential financial conflicts of interest could lead to the following: 1) Prior to publication, article rejection, or 2) Post-publication, sanctions ranging from, but not limited to, issuing a correction, reporting the inaccurate information to the authors' institution, banning authors from submitting work to ASN journals for varying lengths of time, and/or retraction of the published work.

Name: David C. Wheeler
Manuscript ID: CJASN-2025-000773R2
Manuscript Title: Effects of dapagliflozin on CKD progression in patients with different rates of eGFR loss
Date of Completion: July 29, 2025
Disclosure Updated Date: May 28, 2025
