# Supplementary material for: Effects of Dapagliflozin on Progression of CKD According to Different Rates of Pretrial eGFR Loss
Source: Clin J Am Soc Nephrol. 2025 Sep 9;20(11):1527–35. doi: 10.2215/CJN.0000000810 (PMC12614848; doi:10.2215/CJN.0000000810)

Supplementary Material

Supplementary Table 1: Baseline characteristics stratified by treatment allocation and pre-trial eGFR trajectory

	**Dapagliflozin 10 mg**			**Placebo**			**Overall DAPA CKD cohort (N=870)**	**DAPA-CKD**
**Characteristics**	**<−5 n=239**	**≥−5 to <−1 n=116**	**≥−1 n=57**	**<−5 n=248**	**≥−5 to <−1 n=114**	**≥−1 n=96**		**(N=4304)**
Age, years, mean (SD)	61.9 (12.3)	61.8 (12.8)	62.2 (12.7)	62.0 (12.0)	63.1 (12.1)	62.6 (12.3)	62.2 (12.3)	61.8 (12.1)
Sex, female, n (%)	73 (30.5)	33 (28.4)	15 (26.3)	62 (25.0)	30 (26.3)	30 (31.2)	243 (27.9)	1425 (33.1)
Systolic BP, mmHg, mean (SD)	136.7 (18.4)	136.9 (17.8)	133.4 (16.7)	137.5 (18.0)	135.1 (16.9)	138.8 (18.5)	136.8 (17.9)	137.1 (17.4)
Diastolic BP, mmHg, mean (SD)	76.1 (11.4)	78.6 (11.1)	75.1 (10.2)	76.2 (10.7)	77.1 (10.4)	77.5 (10.8)	76.7 (10.9)	77.5 (10.5)
Weight, kg, mean (SD)	83.1 (21.0)	84.3 (20.5)	82.4 (22.5)	81.8 (21.5)[*]	80.6 (19.6)	84.3 (20.5)	82.6 (20.9)	81.7 (20.5)
HbA1c (%), mean (SD)	7.0 (1.5)	6.6 (1.4)	6.7 (1.2)	6.9 (1.5)	6.6 (1.4)[†]	6.8 (1.5)[‡]	6.8 (1.5)	7.1 (1.7)
eGFR, mL/min/1.73m^2, mean (SD)	40.4 (11.3)	37.9 (10.9)	44.0 (12.1)	39.1 (10.9)	37.3 (9.6)	40.8 (11.3)	39.6 (11.1)	43.1 (12.4)
Pre-trial eGFR slope, mean (SD)	−10.5 (4.5)	−3.1 (1.0)	2.2 (1.7)	−10.0 (4.5)	−3.2 (1.1)	2.5 (1.5)	−6.1 (6.1)	n/a
Hemoglobin, g/L, mean (SD)	127.0 (16.0)	131.8 (17.6)	130.6 (20.1)	124.1 (16.8)	131.0 (18.2)	130.6 (16.9)[‡]	128.0 (17.3)	128.3 (17.1)
UACR, mg/g, median (IQR)	1090.5 (481.5, 2273.0)	993.8 (509.8, 1997.8)	1067.0 (623.0, 1543.0)	1231.2 (588, 2512.0)	769.0 (460.8, 1487.5)	785.5 (457.2, 1426.1)	1023.2 (519.8, 1982.8)	949.3 (477, 1885)
Cardiovascular disease, n (%)	92 (38.5)	40 (34.5)	14 (24.6)	89 (35.9)	43 (37.7)	37 (38.5)	315 (36.2)	1610 (37.4)
Heart failure, n (%)	25 (10.5)	10 (8.6)	4 (7.0)	19 (7.7)	13 (11.4)	9 (9.4)	80 (9.2)	468 (10.9)
Previous medication, n (%)								
ACEi, n (%)	73 (30.5)	41 (35.3)	12 (21.1)	62 (25.0)	27 (23.7)	38 (39.6)	253 (29.1)	1354 (31.5)
ARB, n (%)	162 (67.8)	74 (63.8)	43 (75.4)	177 (71.4)	87 (76.3)	58 (60.4)	601 (69.1)	2870 (66.7)
Diuretics, n (%)	117 (49.0)	50 (43.1)	26 (45.6)	111 (44.8)	49 (43.0)	52 (54.2)	405 (46.6)	1882 (43.7)

Statins	167 (69.9)	82 (70.7)	48 (84.2)	160 (64.5)	90 (78.9)	68 (70.8)	615 (70.7)	2794 (64.9)
Antithrombotic agents	119 (49.8)	54 (46.6)	26 (45.6)	125 (50.4)	58 (50.9)	45 (46.9)	427 (49.1)	2042 (47.4)

*n=247; †n=113; ‡n=95; §n=246. ACEi, angiotensin-converting enzyme inhibitor; ARB, angiotensin-receptor blocker; BP, blood pressure; eGFR, estimated glomerular filtration rate; IQR, interquartile range; SD, standard deviation; UACR, urinary albumin-to-creatinine ratio

Supplementary Table 2: On-treatment total eGFR slope trajectories by pre-trial enrollment eGFR slope trajectories.

	N	eGFR slope (mL/min/1.73m^2/year) Placebo	eGFR slope (mL/min/1.73m^2/year) Dapagliflozin	Difference eGFR slope (mL/min/1.73m^2/year)	p-interaction
Total	870	−4.1 (0.2)	−2.8 (0.2)	1.3 (0.7, 1.9)	
UACR, mg/g					0.06
>1000	444	−5.8 (0.3)	−3.8 (0.3)	1.9 (1.2, 2.7)	
≤1000	426	−2.5 (0.3)	−1.6 (0.3)	0.9 (0.1, 1.6)	
Pre-trial eGFR, mL/min1/.73m^2/year					0.001
<−5	383	−4.7 (0.3)	−2.6 (0.3)	2.2 (1.4, 2.9)	
≥−5	487	−3.4 (0.3)	−3.1 (0.3)	0.3 (−0.6, 1.1)	
UACR and pre-trail eGFR					0.12
UACR >1000 and eGFR slope <−5	215	−6.5 (0.3)	−3.7 (0.4)	2.9 (1.9, 3.8)	
UACR ≤1000 and eGFR slope <−5	272	−2.4 (0.4)	−1.3 (0.4)	1.1 (−0.0, 2.1)	
UACR ≤1000 and eGFR slope ≥−5	211	−2.6 (0.4)	−2.0 (0.4)	0.6 (−0.5, 1.7)	
UACR >1000 and eGFR slope ≥−5	172	−4.6 (0.4)	−4.1 (0.4)	0.5 (−0.7, 1.7)	

eGFR, estimated glomerular filtration rate; UACR, urinary albumin-to-creatinine ratio

Supplementary Figure 1: Between group differences in on-trial total eGFR slope as a function of the pre-trial eGFR slope

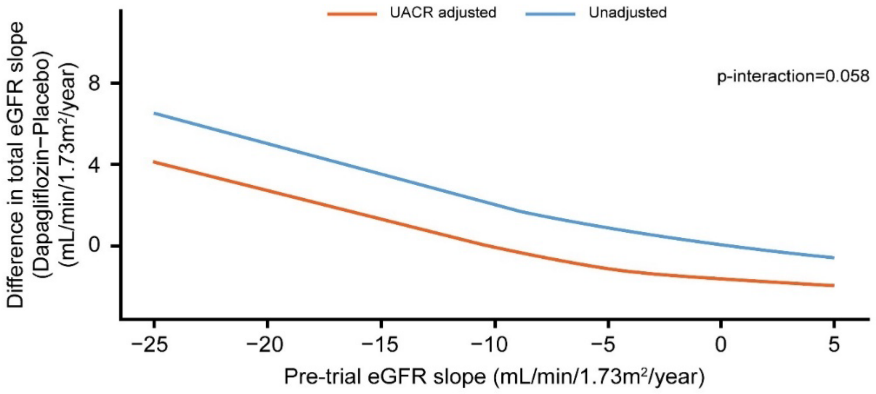

Supplement: Supplementary file 2 [file cjasn-20-1527-s002.pdf]
